# Contested science communication: Representations of scientists and their science in newspaper articles and the associated comment sections

**DOI:** 10.1177/09636625251325453

**Published:** 2025-03-17

**Authors:** Katrine K. Donois, Lewis Goodings, Mick Finlay, Nicola Gibson

**Affiliations:** Anglia Ruskin University, UK

**Keywords:** interaction experts/public, media and science, media representations, public understanding of science, representations of science, science attitudes and perceptions, science communication, scientific controversies, social representations

## Abstract

This qualitative study uses inductive thematic analysis to investigate how journalists and their readers perceive scientists. The data-driven approach was applied to 84 articles (reporting on the contested science issues of climate change, vaccines, or genetically modified organisms (GMOs)) and their associated comment sections. Two dominant groups were observed: the pro-science group (consisting of commentators and journalists) and the contra-science group (nearly exclusively commentators). The identified themes show that both groups represent scientists and their science in a particular and similar way across the three contested science topics. These representations are used to justify both support and opposition (e.g., each group refers to scientists’ motives; however, they express this theme differently by either describing scientists’ actions as born out of a desire to help or out of arrogance). Understanding how non-experts perceive scientists could help improve science communication, which may be the first step toward decreasing societal polarization over contested science.

## Introduction

A major challenge facing science communication is the current polarized environment in which some people endorse science while others routinely disregard or reject scientific evidence (Rutjens et al., 2018; De Cruz, 2020; Rekker, 2021). This trend has profoundly changed how scientific information is received or used (Olson, 2018) and given rise to so-called contested science, which refers to science associated with political or religious ideology-sensitive issues such as climate change, GMOs, or vaccines (Said et al., 2021). In moments when science communication is already of a contested nature, the role of the Internet seems to add further opportunity for confusion and contestation in citizens’ communication and decision-making with its near-unlimited, instant, and often unregulated access to information (Kozyreva et al., 2020). This highlights the need for psychological science to investigate users’ construction of scientists and how it affects active decisions about science in light of the wealth of digital information available.

In 2015, Kahan (2015) argued that the world is confronted with a “science communication paradox” (p.1.) because never have citizens been more educated and had easier access to information; yet, never has public disagreement on science been so ubiquitous. These rising levels of “motivated reasoning” and the prioritization of personal beliefs and ideologies over scientific explanations have received much attention, and researchers have concluded that polarized opinions are not necessarily a byproduct of information deficits, as previously believed (Nyhan and Reifler, 2019; Sturgis and Allum, 2004). Instead, prior beliefs affect how information is interpreted and understood (Fryer et al., 2019), meaning that people tend to view new information within the context of their existing views on a subject (Zhou and Shen, 2022). For example, Kahan et al. (2017) suggested that people tend to form beliefs that either reflect their ideological identity (i.e., unconsciously favored conclusions) or are based on scientific evidence, with the latter more likely to happen if there is no conflict between the individual’s identity and the science. Kahan proposed that to make science communication more efficient, communicators should avoid putting their audience in a position where they would have to make a hard choice between the science presented and their ideological identity. However, as conflicts between beliefs and science are central to contested science, these recommendations are difficult to put into practice.

Furthermore, how scientists and their science are represented has yet to be investigated in the context of affective polarization, which is the tendency of partisans to dislike and distrust people from another political party. For example, Druckman et al. (2020, 2021) found that policy differences between parties are not only due to contrasting values or policy preferences but also affected by dislike or distrust of members from another party (i.e., partisan animus). The latter is relevant to science communication because, according to Rekker (2021, p.352), when it comes to rejecting science, affective polarization can take two forms: psychological science rejection and ideological science rejection. The former refers to disregarding scientific evidence inconsistent with one’s political identity, and the latter relates to adopting a political ideology that specifically contests the science at hand. Affective polarization and partisan animus, concepts from political science, can, therefore, affect acceptance or rejection of science. To date, this has received little attention despite emerging research showing that the public’s attitudes and actions during the pandemic were impacted by affective polarization (Druckman et al., 2020).

Research has also shown a correlation between anti-science stances and a general lack of trust in science in general. For example, Eichengreen et al. (2021) examined the impact of global epidemics (since 1970) on individuals who lived through such an event (when they were between the ages of 18 and 25 years), and they found that such an experience did not affect confidence in science or scientists. This finding was supported in 2023 (the same year the World Health Organization declared an end to the global COVID-19 pandemic (WHO, 2023) when the State of Science Index reported that 33% of their respondents answered that they were skeptical of science (State of Science Index: Global Report 2023)). This number has not changed since 2019 and illustrates how, even with global events like the pandemic, there is distrust of science or scientists, which has become embedded in the everyday understanding of new pieces of information. Furthermore, the contested nature of science is at the heart of this continuing distrust in scientists and their science.

The current literature shows that societal polarization over contested science is complex and multifaceted; however, a commonality of the studies exploring this issue is the tendency to describe the conflicting discourses about science as debates between opposing sides (Weisberg et al., 2021). One of the places such debates can be found (and where competing accounts and conflict emerge) is in newspaper comment sections in which readers react to the content of an article. However, despite the existence of a broad range of work on the role of the media in creating polarization (see, e.g., Dahl, 2015; Calice et al., 2023), the readers’ comments have not been investigated in those who focus on newspaper articles (see, e.g., Chinn et al., 2020; Hart et al., 2020; Feldman et al., 2017; Pjesivac et al., 2020). The latter has created a gap in our knowledge, particularly regarding how readers portray scientists and their science in these online spaces. In addition, research concerning how journalists and their readers view scientists working in and communicating about contested science remains scarce as most research has focused on trust in expertise and science (Lobato and Zimmerman, 2018). Finally, qualitative investigations are particularly underrepresented within the study of science communication (Lakomý et al., 2019).

## Objective

This study examines how scientists and their research are portrayed in newspapers and associated comment sections. Understanding this could help improve science communication, which may be the first step toward decreasing societal polarization over contested science (Jucan and Jucan, 2014; Rekker, 2021). This study adopts a qualitative perspective to closely examine the ways that journalists represent scientists when reporting on contested science issues. In addition, it will explore how the scientists and their science are portrayed by the community of readers in the comments section below the article. Thus, the analysis addresses the following research question: How are scientists and their science represented in newspaper articles and comment sections about contested science issues, such as anthropogenic climate change, genetically modified organisms, and vaccines?

## Method

Data were collected in 2021 and focused on digital news articles published between 2015 and 2021. These articles were from four high-circulating newspapers representing a broad political spectrum: The Guardian and The New York Times (both liberal) and The Times and The Washington Post (both conservative). These British and American newspapers were selected because they are widely circulated in their respective countries and read worldwide. As this study is positioned within contested science, the chosen newspaper articles cover either climate change, GMOs, or vaccines. It is important to acknowledge that these are not the only contested science topics; however, they tend to be discussed in similar terms in the UK and the US, and studies frequently use data from both countries (see, e.g., Dahl, 2015; Krange et al., 2021; Lefere et al., 2023; Mellish et al., 2020).

The news articles were obtained from the archives found on the homepages by entering specific keywords/search terms (i.e., GMO(s), genetically modified organism(s), gene editing, genetic engineering, climate change, global warming, vaccine(s), anti-vaxxer(s), and anti-vax). Gene editing and genetic engineering were also included because they are often used interchangeably with GMOs by non-experts (Foht, 2016; Muringai et al., 2020). In order to get a broad sample, all types of articles were included (e.g., news stories, editorials, and columns). “Letters” from readers were excluded because they were not written by journalists. This produced 151,041 initial results. The articles were then filtered by the following criteria: First, the articles had to directly relate to the research question and cover one of the contested science areas (GMOs, climate change, and vaccines). Those that did not focus on these contested areas were discarded. Only articles with more than seven posts in the comment sections were considered for this study (as this indicated reasonable evidence of impact). The articles where scientists and the contested science areas were not mentioned in the associated comment section were removed. The final sample contained 84 articles and 25.121 associated comments, which were taken forward for analysis as they offered a rich and detailed data set. This is in line with or above the typical amount of data considered by a thematic analysis (TA). For example, Bierbaum (2021) selected 12 articles per newspaper (20 newspapers in total) for the analysis of news coverage addressing the impacts of public school closure. Another study investigating bariatric surgery in adolescents by Lefere et al. (2023) collected 38 articles in total (or 1–2 articles per newspaper), and Koerber et al. (2017) examined 26 articles (or 3–4 articles per newspaper) about workers with hearing loss. The average number of articles collected from newspapers in the above-mentioned three studies is six.

A total of 25,121 comments constitute a considerable data set. As is common in the TA of large online data sets, only a selection of posts was taken forward for analysis (e.g., King and McCashin, 2022), partly because many comments were not suitable for this study (e.g., spam, incomprehensible, off-topic, or composed mainly of emojis). Thus, a keyword search was first conducted to identify the areas of interest. These keywords were “science,” “scientist(s),” “researcher(s),” “expert(s),” and “expertise.” All comments containing one or more of these keywords were collected. Some comments were posted and re-posted several times; therefore, the re-posts were discarded. As a result, 257 comments were selected as they offered a rich and detailed data set that enabled the research question to be answered.

## Analysis

A qualitative methodology using TA was employed. TA is the process of identifying themes in qualitative data to understand a particular context or phenomenon (Braun and Clarke, 2022). TA involves coding and theme development, which “are assumed to be subjective and interpretative processes” (Terry et al., 2017, p.7). Some of the advantages of using TA include the useful summarization of key features within a large body of data and that the results are generally considered accessible to non-experts because TA “offers a ‘thick description’ of the data set [while highlighting] similarities and differences across the data set” (Braun and Clarke, 2006: 97). It is important to note that qualitative research, such as TA, does not tend to refer to statistical-probabilistic generalizability (to a whole population), but findings are often still “transferable” to similar groups or situations as long as the source of the data is explicitly described, and such assumptions are made with care (Byrne, 2022). Thus, as TA aims to present a coherent narrative with contextual information based on a detailed account of a rich data set (Herzog and Kelly, 2023), it was considered an appropriate method for this inductive (data-driven) study.

**Figure 1.**
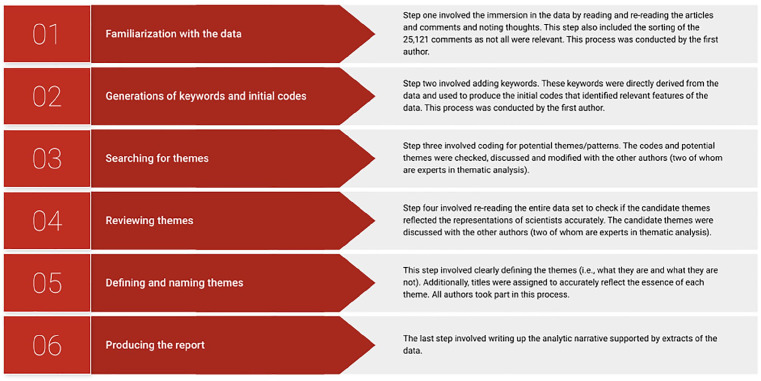
This figure shows the six steps that guided the meticulous processing of the qualitative data, which in turn serves to “enhance the rigor of the research process and the depth of research findings” (Naeem et al., 2023: 2).

**Figure 2.**
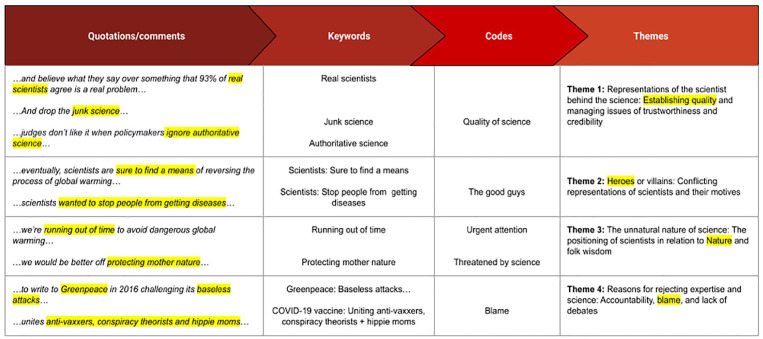
This figure shows an extract of the inductive analytical process and serves as an example of how Braun and Clarke’s (2006, 2022) methodological approach and Naeem, Ozuem, Howell, and Ranfagni’s (2023) step-by-step process of thematic analysis were applied to the data.

Six phases were followed. Figure 1 shows the analytic process (see also supplemental material), which included familiarization with the data, generations of keywords and initial codes, searching for themes, reviewing themes, and finally, defining and naming themes before producing the report (Braun and Clarke, 2006, 2022; Naeem et al., 2023). Initially, the articles and posts were read and re-read in order to note keywords and codes and identify potential themes. The second level of analysis (see Figure 2) involved reviewing these initial codes and naming the first tentative themes. The research questions informed this process (i.e., by checking that these initial codes and themes were directly related to the research question). Finally, quotations from the comment sections and the articles that illustrated the overarching themes were compiled, and the themes were reviewed and named.

It is important to note that TA “does not rely on, for example, frequency counts to indicate the importance of coded data” (Jowsey et al., 2021: 473). Nevertheless, adverbs serving as quantifiers (Lewis and Keenan, 1975) have been added to highlight whether the described perceptions are frequently or less frequently present in the data set.

## Results

**Figure 3.**
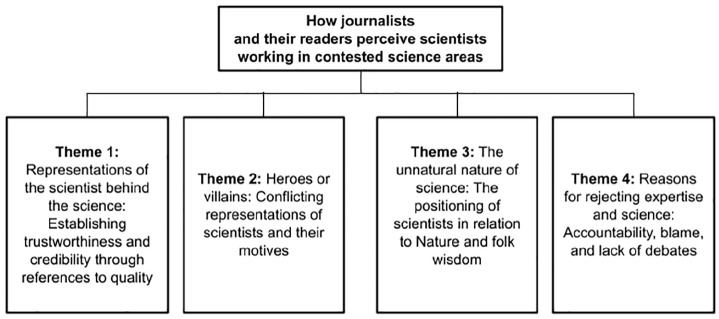
A map of the four themes.

This section presents the four most prevalent themes (see Figure 3) identified in the newspaper articles and the associated comment sections. The results below include quotes selected to illustrate the identified themes. All extracts are presented in their original formatting, including errors in punctuation and spelling. We have removed the names of the journalists and commentators.

### Theme 1: Representations of the scientist behind the science: Establishing trustworthiness and credibility through references to quality

In the articles and comments sections, there is a frequent reference to “real science” or “junk science” (as in poor or pseudo-science). This represents scientists as either trustworthy and credible or the opposite. The following three posts about vaccinations are examples of this tendency. These posts also show signs of contestation as there are some instances where the comments are accepting of the science but still work to portray the scientist negatively, which, in turn, is presented as the reason for rejecting their science.

Post 1 was posted in response to an article on vaccines, and it begins with a positive description:

Post 1: *“Vaccinations are public health. Like clean water. But now public health campaigns are really more about policing private behaviour and contain junk science as well as being specifically exempt from evidence based medicine. These are not people with hoax degrees of the internet, but genuine medical professionals and scientists. Which is what makes them more damaging to the reputation of science: if they use evidence lite junk science on one issue, and with a haughty tone to boot, why are people surprised when they are not always believed when promoting vaccination programs?”*

Here, the importance of vaccinations is emphasized. However, public health campaigns are described as containing junk science and being more about telling individuals what to do while disregarding their rights to private behavior. The second part of Post 1 describes how “genuine” scientists use “junk science” accompanied by a sense of superiority, which serves as a rationale for disregarding the science. The following post refers to the Trump administration and how it handled the COVID-19 pandemic:

Post 2: “*I will not get vaccinated until Biden has been in office long enough to appoint real doctors, researchers and scientists to review any vaccine and to start development all over again if necessary*.”

This is an example of also not necessarily being against the science at hand. Instead, the problem is with the trustworthiness of the scientists working under the Trump administration.

Post 3: “*It’s time now for vaccines to be turned into a real science; the current bias, assumption and lack of independence leave them as simply a religious belief*

*The studies are not being designed to find harm; you won’t find what you don’t look for. All studies are not equal, mostly what we see with vaccines is researchers who’ve been paid by industry to play games with numbers and create a pseudo-scientific advert for a vaccine*.”

Post 3 shows how researchers are perceived by some individuals as untrustworthy and not credible. This post is similar to the prior two posts in that it appears as being not against the science per se by implying that the problem is with something else—such as lack of confidence in a president and their administration or, in this case, the design of research studies. Though the three posts are written from different points of view (and the first two appear more pro-vaccines than the last one), they all distinguish between quality or “real” science and untrustworthy scientists or “junk” (pseudo) science.

Below is an example of how a journalist refers to those who oppose the science as “contrarians” while simultaneously calling climate scientists “mainstream.” This shows how journalists also position scientists and their science within a similar dichotomy of “junk” versus “real” science:

if the contrarians are right, the 2°C resulting global warming would represent significantly less severe climate change consequences than if mainstream climate scientists are right.

This quote illustrates how journalists reference quality. In other words, contrarians’ claims are pinned against those of mainstream climate scientists, who, in turn, are described as being right.

References to quality in order to establish or dissolve the trustworthiness and credibility of scientists and their science are observed in all three contested science topics. The focus on “real” science is primarily seen in comments but also appears regularly in the articles, whereas references to “junk science” are observed almost exclusively in the comments.

### Theme 2: Heroes or villains: Conflicting representations of scientists and their motives

Those who are “for” the science at hand often describe scientists, their science, or scientific developments in terms that resonate with the idea of “the good guys,” who are trying to, for example, save people, prevent disease, or fight starvation. The following is a comment to a Washington Post article reporting on “golden rice” (a genetically modified rice developed to mediate the vitamin A deficiency syndrome):

Thankfully they have finally seen the light on a very beneficial and really quite straight forward GMO product. . . . GMOs developed with strict guidelines of course, will save the human species.

This comment shows GMOs described as something that can save and help people, often by preventing tragedies from happening. Such prevention attempts are referenced in all three contested science areas. For example, vaccines are described as an attempt to keep people from succumbing to diseases (*“Scientists wanted to stop people from getting diseases, so they created vaccines.*”). Regarding climate change, scientists are perceived as trying “*to prevent more lives being lost to extreme weather*.” Finally, scientists are perceived as working for the common good (or, as one commentator wrote in the Guardian’s comment section: “*My faith in highly educated, dedicated scientists researching and treating for the common good remains strong*”).

In contrast to those who perceive scientists as the good guys, those against the science at hand tend to describe them as arrogant, playing God, and suffering from hubris. In addition to engaging in dangerous science, scientists are also perceived as lacking ethics training and morality. Below is an example from the Washington Post of scientists represented as arrogant and lacking “native intelligence”:

What I find mind-boggling is the disconnect between science and the rest of us. I’m not a transplant surgeon, genetic researcher or a scientist, but I’ve worked alongside them for years and have found too few willing to drop their bloated arrogance and recognize there’s a difference between having native intelligence and having smarts.

Here, the commentator explains that they are not a scientist or researcher, but they have an in-depth knowledge of what scientists are like since they worked with them for years, and they have found most scientists to exhibit “bloated arrogance” and an unwillingness to recognize that knowing a lot of facts is not the same thing as understanding the deeper implications of applying them. Accordingly, this post shows how scientists are described as exuding an exaggerated sense of self-importance (i.e., arrogance).

The following post shows scientists described as contributing to problems facing humanity and creating unnecessary or harmful products out of “human arrogance” and by “playing god.”

Science is not inherently good. Science created atomic weapons. We need fewer scientists and more philosophers! (Says the liberal arts major . . . )

There is absolutely no need for GMO foods. This is human arrogance, playing god. Humanity managed to grow and thrive for millennia without GMO foods.

Scientists are also seen as suffering from hubris: “*Human hubris. It’s just a matter of time before the law of unintended consequences takes over.*” This post highlights how scientists are frequently perceived as not being in control of their science, which will result in “unintended consequences” or dangerous science. The notion of “dangerous science” is exemplified in the following post from the Guardian, where the commentator highlights the idea that scientists are preoccupied with a result and not consequences: “*Basically this article is saying it doesn’t matter how lethal, dangerous, untested, or unethical the means is,.if it feeds more people it’s OK to sell.*” This post also shows that references to “unethical” science or scientists are common. For example: “*Many researchers and scientists a very intelligent and very analytical but divorced from the ethics a lack empathy.*” This described lack of ethics and empathy shows how some view scientists as intelligent yet as figures who do not consider the moral implications of their work.

In contrast to those who perceive scientists as wanting to help or solve problems, some describe them as the bad guys taking pleasure in hurting or experimenting on people: “*We have become guinea pigs for food scientists, who seem to delight in producing ‘frankenfoods’*.”

The tendency to draw on Hollywood when describing scientists or their science is also found in the newspapers. For example, in a New York Times article about gene drives (technology that helps spread a gene through, e.g., populations), a journalist writes that

Hollywood, of course, isn’t a precise litmus test for how a new technology is likely to be received by the public. But it’s also not a bad approximation. Like screenwriters, most of us tend to gravitate toward the more extreme examples of a technology’s potential, its ability to save the world or to destroy it.

This quote shows that, in connection with meaning-making, non-experts tend to draw on movie depictions of science and scientists.

The conflicting representations of the motives of scientists and their science are observed in all three contested science areas. Portrayals of scientists as working for the common good, collaborating, and being motivated by a want or desire to save, help, or prevent are predominantly seen in the articles, though also found in the comments. In contrast, portrayals of scientists as arrogant, playing God, suffering from hubris, engaging in dangerous science, and lacking ethics training and morality are primarily observed in comments to GMO and vaccine-related articles; however, the tendency is also present (to a lesser extent) in the climate change articles and comments.

### Theme 3: The unnatural nature of science: The positioning of scientists in relation to Nature and folk wisdom

Journalists and commentators repeatedly evoke the concept of Nature throughout the three contested science areas.

In the group “for” the science at hand, Nature tends to take on either a negative or positive form. In the negative form, threats from Nature are described, such as floods (climate change) and diseases (vaccines and GMOs); however, this is seen primarily in the articles rather than in the comments and is something journalists tend to do when they provide background or context for their story. For example: “*By the 1990s, scientists had a deep understanding of the future risks of a warming world. By the 2010s, researchers could show how the extreme heat waves, droughts and floods now unfolding were influenced by climate change.*”

Threats from Nature are also portrayed as needing urgent attention (“[C]*limate crisis is one of our most urgent crises*”) and as a race against time: “*climate scientists are still right, and we’re running out of time to avoid dangerous global warming.*”

In these examples, threats are due to human actions, and therefore, scientists are trying to protect a “good” Nature from the impact of anthropogenic climate change manifested through a “bad” Nature. This dualistic view, in combination with urgency, is also present in the other contested science topics. For example, scientists are portrayed as racing to protect crops from diseases (“*Scientists scramble to stop bananas being killed off. British firm races to produce bananas resistant to fungus sweeping global plantations.*”) or to develop a vaccine for a life-threatening disease to save lives (“*scientists race to create a vaccine for the deadly coronavirus* . . .”). Both examples portray scientists as pressed for time trying to protect or save a “good” nature (bananas or people) from a “bad” nature (fungus or the coronavirus).

In the group “for” the science at hand, Nature is also frequently presented in a positive form. Here, scientists are portrayed as having worked in tandem with Nature for decades or as using Nature as a tool to discover ways to, for example, optimize or improve farming or health. This tendency is observed predominantly within the articles rather than the posts. For example: “*Gene editing allows us to give mother nature a helping hand.*”

In contrast, those commentators who are against the science mentioned in the articles bring up old wisdom or Indigenous people as an alternative to scientific developments or solutions:

Why is Phoenix not consulting the Native Americans or the native Arabs to understand how they survived the heat in the old days? Native people were pretty smart in how they lived in their environments. Modern scientists may not have all answers. Need old wisdom too!

Nature is also described as knowing better: “*I guarantee you Nature has far greater insight into human nutritional needs than anything that these smug scientists can ever come up with. She’s been working in her laboratory for eons to get it just right.*”

Other comments present science as a threat to Nature:

nature’s ‘original’ and proven seeds deserve protection from our well-meaning but potentially damaging ‘leaps’ over natural genetic drift. There may be no way to go back to safe crops if humans are wrong about GMO impacts.

By skipping natural processes and interfering with Nature, science is also described as hurting people:

The vulnerability of very young babies to measles today is the direct outcome of the prolonged mass vaccination campaign of the past, during which their mothers, themselves vaccinated in their childhood, were not able to experience measles naturally . . . and establish the lifelong immunity that would also be transferred to their babies and protect them from measles.

Finally, Nature is portrayed as threatened by greed and scientific fixes in which the scientists responsible are represented as not caring about the health or environmental impacts of their developments:

If people are enthusiasts about scientists playing with Nature just to make more money, then they should evaluate the real consequences of giving up food production. . . . When money is the aim, no health nor environmental issues pose any concern to the developers.

### Theme 4: Reasons for rejecting expertise and science: Accountability, blame, and lack of debates

This theme shows how those for the science at hand frequently tend to blame activist groups, such as “hippie moms” or “Greenpeace,” for influencing the acceptance or rejection of scientific recommendations or communications. The latter is found in all three contested science areas. The following quote from the Washington Post shows how Greenpeace is described as engaging in “baseless attacks” on the GMO produce, “golden rice”: “*The green campaign against beta-carotene-rich ‘golden rice’—which could save hundreds of thousands of poor and undernourished children from blindness and death caused by vitamin A deficiency—prompted more than 100 Nobel Prize winners to write to Greenpeace in 2016 challenging its baseless attacks.*”

The comment sections also mention the influence of Greenpeace. For instance, one reader of The Guardian writes, “*GM can be a good and useful thing.* ()*The problem is the knee jerk reaction of some people and international pressure groups such as Greenpeace*.” Those in favor of alternative medicine, anti-vaxxers, and conspiracy theorists are also presented as reasons for science rejection: “*Prospect of a coronavirus vaccine unites anti-vaxxers, conspiracy theorists and hippie moms in Germany.*” The latter is an example from a journalist who points out that anti-vaccine sentiments are related to “*alternative and holistic medicine traditions*” (e.g., homeopathy and anthroposophical medicine). Though readers and journalists agree that these groups or movements influence acceptance of expert recommendations, some also point to a lack of responsibility for one’s fellow citizens and selfishness as an explanation for science rejection:

we all have some responsibility for the health of our brethren. Older people, people undergoing chemotherapy, people suffering from heart issues, diabetes, COPD, Liver and Kidney failure, etc. will likely die if infected. Do these people even care? This is the direct result of a “me” mentality that says that we have no responsibility for anyone else.

Contrarians and doubters are also blamed (the latter is found in all three contested science areas):

That’s why you make up your science and why you claim that you have access to data that is kept hidden from the rest of humanity. That’s why you pretend to be the expert yet won’t have your contrarian opinions tested by the powers that be.

Here, a reader of the Times describes how contrarians pretend to be experts, invent (bad) science, and claim access to hidden data while refusing to have their opinions tested. The Media and the Internet are often blamed for circulating the ideas of these types of groups: “*The media is largely to blame for anti-vaxxers gaining ground*” or *“These outbreaks* [are] *due to Internet-fueled, debunked conspiracy theories claiming vaccines cause autism or other health risks*.”

In contrast, those against the science at hand frequently state that they have a right to choose whether or not they will accept scientific recommendations (this is seen primarily in regard to vaccines or GMOs) because “[n]*o scientist has the right to take this choice from another human*.” In addition to emphasizing the right to choose, the contra-science group also perceives money and business as influencing science journalism, governments, and institutions, leading to biased science or scientists (this is found in comments to all three contested science areas).

Post 1: “Then you have the dire quality of science journalism, repeating press releases uncritically effectively helping a PR campaign. In so many studies that are splashed over the media, the flaws are easy to see once you know what to look for. The influence of money and business is everywhere.”

Post 2: “I don’t trust that scientists have freedom of thought either, because our governments are so overrun with private interests.”

Another reason to reject science is a perceived lack of accountability, meaning that when scientists make mistakes, they do not admit it or are not held accountable. This is exclusively found in the comment sections. The notion of not taking responsibility for errors is frequently referenced, for example:

Scientists told us a high carb, low fat, low salt diet was good for us, and that trans fats were superior to animal fats.

They told us that DDT accumulated in the food chain and were killing eagles.

Scientists told us CFCs were creating a hole in the ozone layer.

All wrong, but scientists have never refuted their errors.

Finally, the two groups have different takes on debating science. For example, the pro-science group regularly highlights that people should accept what scientists communicate because they are the experts. “*We have to start believing experts in fields in which we have little knowledge*.” Though the pro-science group advocates for following scientific recommendations (“*it is ok to listen to experts, why wouldn’t you?*”), journalists rarely state explicitly that people should listen to experts. Instead, they tend to add quotes making this point; for instance, in an article from The Times about climate change, President Biden is quoted,

Mr Biden also sought to link Mr Trump’s rejection of scientific warnings about climate change with his handling of the coronavirus. “We know he won’t listen to the experts or treat this disaster with the urgency it demands, as any president should do during a national emergency.”

In contrast, those who disagree with the science at hand frequently state that they do not want to listen to experts because the media or experts are not listening to them: “*There is only one constituency whose opinions are being surveyed in this article*: ‘*plant scientists.*’ *Apparently the concerns of the rest of us about the impact of GMOs aren’t important enough for the Guardian to mention.*” and ( “*in this case the* ‘*experts*’ *are not even addressing my concerns*!”). This goes hand-in-hand with a perceived lack of debate: “*People who don’t understand science should be allowed to discuss it. After all it can be remarkably hard to talk to a scientist about things.*” However, debates are also perceived as a positive thing that, for example, could rebuild some trust:

Well who can you trust? Think. If you have a transparent platform where experts, academia and the public get involved—it’s reasonble to state that that’s more transparent and inclusive for intellectual discourse. Crap stuff will get voted down. Good stuff get voted up. Meritocracy wins. Trust is gained and we can’t point the finger at others as we all get to learn and contribute.

Theme 1 shows how the trustworthiness and credibility of scientists tend to be repeatedly challenged. However, the final example in Theme 4 shows that in the contra-science group, many are calling for more debates between scientists and non-experts—which, in turn, is also perceived as a way to gain trust, foster learning, and allow contributions from everyone. In the above post, the commentator suggests building an inclusive platform where scientists and the general public can discuss, explain, and ask questions. This shows that transparency and public involvement through debates are perceived to affect trust in scientists working with contested science. However, it depends on the perceived willingness of scientists to answer questions and engage in these debates or, as the same commentator wrote, “*if of course you are ready to get inside the ring and be part of the change.*”

## Discussion

This study examines how journalists and their readers represent and perceive scientists (who work in contested science areas). Four themes were observed in relation to the research question. Theme 1 illustrates how quality is established and how it affects trustworthiness and credibility. Theme 2 describes the contradictory representations of scientists and their motives. Theme 3 displays how scientists are thought of in relation to Nature and folk wisdom. Theme 4 captures perceived reasons for rejecting or accepting science and expertise. These themes illustrate the key recurring patterns in the data and identify issues embedded in the everyday discussions of contested science, and will now be discussed in more detail.

Theme 1 shows how the concepts of “real” and “junk” science are central to how contested science is discussed. The data suggest that these concepts can be used to weaken or dismiss the quality of the evidence presented by either group, a finding that is related to research showing how rejecting scientific evidence tends to be framed as scientific skepticism stemming from experts within scientific communities (Al- Rawi et al., 2021; Jacques, 2012). More importantly, however, many commentators who criticize the credibility of scientists (or the quality of their science) appear to be not necessarily anti-science in general. This finding is important to science communication because, even though more research is needed, it implies that anti-science views are more nuanced than commonly perceived (O’Connell et al., 2020; Philipp-Muller et al., 2022).

Two dominant groups (i.e., those “for” and those “against” the contested science at hand) are observed, and these two groups have contradictory views of scientists and their science (this may be due to the newspapers selected as differences are likely to be found in other outlets, such as tabloids). The pro-science group consists of commentators and journalists, whereas the contra-science group nearly exclusively comprises commentators.

The former adds to the debate about the role of journalists in creating controversies over science. Studies have suggested that journalists play a part in manufacturing doubt within the public (Stocking and Holstein, 2009); however, according to Ward (2019), the differences between each media outlet in how they cover the same topic and the varying visibility of the diverse sources used make it difficult to reach any conclusions. Nevertheless, this study found that nearly all the journalists were positive toward scientists and their science. Specifically, all were pro-vaccines, no one questioned climate change, and 17 articles out of 21 presented the science of GMOs in a positive light. This suggests that, in these four “broadsheet” newspapers, journalists appear to be positive toward scientists and their science (though more research is needed to determine if this applies to all types of contested science). It also supports recent research suggesting that the relationship between scientists and journalists may not be as strained as previously described (Moorhead et al., 2022; Peters, 2013).

Researchers have proposed that incorrect or misrepresented science in the media tends to orbit around exclusion from information due to, for example, journal paywalls that consequently limit journalists’ access to research (see, e.g., Benson, 2019; Finch et al., 2014). In addition, Sumner et al. (2016) found that exaggerated or simplistic news about science could be traced back to university press releases (often approved by the researchers themselves). Relevant to Themes 1 and 2, the finding that journalists in these newspapers tend to be positive toward scientists and their science highlights the existence of an opportunity for scientists to improve the quality of the information relayed to the public via the media, by collaborating more with and providing better information to journalists (Dempster, 2020).

Theme 3 shows that some people pit scientists against Indigenous or old wisdom as another way of discounting expertise. This strategy has been previously observed within environmental movements that emphasize “indigenous perspectives on the relationship between human beings, non-human nature, and culture” by affirming “the ‘sacredness of Mother Earth’” (Lee, 1992; Schlosberg, 2013: 39). Such argumentation has also been found by Motta and colleagues (2018; 2020) in connection with public support of health policies. In addition, Theme 2 shows that scientists are frequently described as “mouthpieces” for industry or government, meaning that Industry-supported research appears to lack credibility and trustworthiness. Therefore, understanding that some people perceive scientists as working against Nature or as instruments of profit-seeking private enterprises could help better prepare science communicators for public debates.

Theme 4 shows that the reasons given for rejecting science or expertise are not the same in the pro- and contra-science groups. For example, the pro-science group blames activists or pretend experts, whereas the contra-science group frequently laments a perceived lack of debate and accountability when scientists are wrong. This finding suggests that inviting questions and debates could be a strategy aimed at lowering polarization over contested science (Kim et al., 2022).

The references to politics made by some commentators point to affective polarization (the inclination of partisans to dislike and distrust people from another party (see Druckman et al., 2021)), meaning that affective polarization may not only be limited to political settings but can also affect the impact of science communication. Put differently, Hrbková et al. (2024) suggested that politics and society are increasingly perceived as divided between “us” and “them.” Thus, it can be argued that polarization over contested science is also affected by an “us-versus-them” mentality in which groups of like-minded people reject scientific findings based on motivated cognition (i.e., rejecting information or scientific evidence perceived as incompatible or threatening to the group identity, core beliefs, or worldview) (Lewandowsky and Oberauer, 2016; Philipp-Muller et al., 2022; Van Scoy et al., 2022). The latter has implications for science communication because it suggests that for some people, being “against” or “for” science and scientists is part of their identity, which in turn indicates that political or religious ideology and values have much more impact on opinions than standard communications about scientific findings (Nisbet and Scheufele, 2009). More research is needed to identify science communication strategies to counter this trend.

This study has a number of limitations. First, the focus was on the contested science of GMOs, vaccines, and climate change, and though an underlying pattern was observed regarding how scientists and their science are perceived in light of the three topics, more research is needed to see if this extends to other contested issues. In addition, the data came from four broadsheet newspapers in the United Kingdom and the United States, and these journalist-authored articles and comments might differ from those found in other (non-English speaking) countries or types of newspaper outlets (i.e., tabloids).

The sample of commentators in this study does not represent the entire population. It is possible that those who comment on the content of an article are so-called super-participants or highly active participants (Graham and Wright, 2014; Wright, 2018). In 2022, a study showed how a minority (12 people) were responsible for up to 65% of anti-vaccine content on Facebook, Instagram, and Twitter^1^ (Nogara et al., 2022). Another recent study found that 10% of Twitter users were responsible for 80% of all tweets (including original tweets, retweets, and quote tweets) created by U.S. adults in 2019 (Wojcik and Hughes, 2019). Further research is needed to explore if the opinions expressed by this minority divert from or are similar to those who do not engage with comment sections or on social media platforms. Such research is important as it is possible that the influence of these anti-science super-participants has been underestimated (as illustrated by a recent study that showed how some parents tend to assign as much credence to social media posts as they do to expert medical information or recommendations (Calarco and Anderson, 2021)).

In closing, the identified themes show that in these articles and the associated comment sections, those “for” or “against” the contested science of vaccines, GMOs, and climate change construct scientists in a particular yet similar way. These representations are used to justify both support and opposition. Decreasing societal polarization over science is not a simple task, and beliefs about science are hard to change as they “are entangled in our social and political environment, shaped by mass and social media portrayals, and confounded by interpersonal and cultural influences” (Akin and Scheufele, 2017: 25). Thus, awareness of how non-experts represent scientists and their science is important as these representations partly shape and inform the public understandings and beliefs. In extension, such awareness can help improve science communication by, for example, preparing science communicators better for the types of discourses or criticisms they can be confronted with in a non-academic forum.

## Supplemental Material

sj-docx-1-pus-10.1177_09636625251325453 – Supplemental material for Contested science communication: Representations of scientists and their science in newspaper articles and the associated comment sections Supplemental material, sj-docx-1-pus-10.1177_09636625251325453 for Contested science communication: Representations of scientists and their science in newspaper articles and the associated comment sections by Katrine K. Donois, Lewis Goodings, Mick Finlay and Nicola Gibson in Public Understanding of Science

## Notes

1 Twitter changed its brand name to “X” in 2023 (Stokel-Walker, 2023).

## References

[bibr1-09636625251325453] Akin H Scheufele DA (2017) Overview of the science of science communication. Oxford Handbook of the Science of Science Communication 14: 25–33.

[bibr2-09636625251325453] Al- Rawi A OʼKeefe D Kane O Bizimana AJ (2021) Twitter’s fake news discourses around climate change and global warming. Frontiers in Communication 6: 729818.

[bibr3-09636625251325453] Benson R (2019) Paywalls and public knowledge: How can journalism provide quality news for everyone? Journalism 20(1): 146–149.

[bibr4-09636625251325453] Bierbaum AH (2021) News media’s democratic functions in public education: An analysis of newspaper framings of public school closures. Urban Education 56(9): 1485–1519.

[bibr5-09636625251325453] Braun V Clarke V (2006) Using thematic analysis in psychology. Qualitative Research in Psychology 3(2): 77–101.

[bibr6-09636625251325453] Braun V Clarke V (2022) Thematic Analysis: A Practical Guide. New York: Sage.

[bibr7-09636625251325453] Byrne D (2022) A worked example of Braun and Clarke’s approach to reflexive thematic analysis. Quality & Quantity 56(3): 1391–1412.

[bibr8-09636625251325453] Calarco JM Anderson EM (2021) “I’m Not Gonna Put That On My Kids”: Gendered Opposition to New Public Health Initiatives. Socarxiv. Available at: 10.31235/osf.io/tv8zw

[bibr9-09636625251325453] Calice MN Bao L Freiling I Howell E Xenos MA Yang S , et al (2023) Polarized platforms? How partisanship shapes perceptions of “algorithmic news bias.” new media & society 25(11): 2833–2854.

[bibr10-09636625251325453] Chinn S Hart PS Soroka S (2020) Politicization and polarization in climate change news content, 1985-2017. Science Communication 42(1): 112–129. 10.1177/1075547020950735 PMC7447862 38602988

[bibr11-09636625251325453] Dahl T (2015) Contested science in the media: Linguistic traces of news writers’ framing activity. Written Communication 32(1): 39–65.

[bibr12-09636625251325453] De Cruz H (2020) Believing to belong: Addressing the novice-expert problem in polarized scientific communication. Social Epistemology 34(5): 440–452.

[bibr13-09636625251325453] Dempster G (2020) The communication of scientific research in news media: Contemporary challenges and opportunities. Journal of Science Communication 19(03): C06.

[bibr14-09636625251325453] Druckman J Klar S Kkrupnikov Y Levendusky M Ryan JB (2020) The political impact of affective polarization: How partisan animus shapes COVID-19 attitudes. Preprint at Psyarxiv. Available at: 10.31234/osf.io/ztgpn

[bibr15-09636625251325453] Druckman JN Klar S Krupnikov Y Levendusky M Ryan JB (2021) Affective polarization, local contexts and public opinion in America. Nature Human Behaviour 5(1): 28–38. 10.1038/s41562-020-01012-5 33230283

[bibr16-09636625251325453] Eichengreen B Aksoy CG Saka O (2021) Revenge of the experts: Will COVID-19 renew or diminish public trust in science? Journal of Public Economics 193: 104343. 34629566 10.1016/j.jpubeco.2020.104343 PMC8486491

[bibr17-09636625251325453] Feldman L Hart PS Milosevic T (2017) Polarizing news? Representations of threat and efficacy in leading US newspapers’ coverage of climate change. Public Understanding of Science 26(4): 481–497. 26229010 10.1177/0963662515595348

[bibr18-09636625251325453] Finch J Bell S Bellingan L Campbell R Donnelly P Gardner R , et al (2014) Accessibility, sustainability, excellence: How to expand access to research publications. Executive summary. Contributions to Science 10(1): 81–88. 10.2436/20.1501.01.187 24400530

[bibr19-09636625251325453] Foht BP (2016) Gene editing: new technology, old moral questions. The New Atlantis 20: 3–15.

[bibr20-09636625251325453] Fryer RG Jr Harms P Jackson MO (2019) Updating beliefs when evidence is open to interpretation: Implications for bias and polarization. Journal of the European Economic Association 17(5): 1470–1501.

[bibr21-09636625251325453] Graham T Wright S (2014) Analysing ‘super-participation’in online third spaces. In: Cantijoch M Gibson R Ward S (eds) Analyzing Social Media Data and Web Networks. London: Palgrave Macmillan, pp. 197–215.

[bibr22-09636625251325453] Hart PS Chinn S Soroka S (2020) Politicization and polarization in COVID-19 news coverage. Science Communication 42(5): 679–697. 38602988 10.1177/1075547020950735 PMC7447862

[bibr23-09636625251325453] Herzog C Kelly P (2023) Applying thematic analysis to analyse press coverage in cross-country comparative research: A qualitative study protocol. International Journal of Qualitative Methods 22: 16094069231179433.

[bibr24-09636625251325453] Hrbková L Macek J Macková A (2024) How does the “Us” versus “Them” polarization work? Capturing Political Antagonism with the Political Antagonism Scale. East European Politics and Societies. Epub ahead of print 11 January. DOI: 10.1177/08883254231215513.

[bibr25-09636625251325453] Jacques PJ (2012) A general theory of climate denial. Global Environmental Politics 12(2): 9–17.

[bibr26-09636625251325453] Jowsey T Deng C Weller J (2021) General-purpose thematic analysis: A useful qualitative method for anaesthesia research. BJA Education 21(12): 472–478. 34840819 10.1016/j.bjae.2021.07.006 PMC8606608

[bibr27-09636625251325453] Jucan MS Jucan CN (2014) The power of science communication. Procedia-social and Behavioral Sciences 149: 461–466.

[bibr28-09636625251325453] Kahan Dan M (2015) What is the ‘Science of science communication’? Journal of Science Communication 14: 1–12.

[bibr29-09636625251325453] Kahan DM Landrum A Carpenter K Helft L Hall Jamieson K (2017) Science curiosity and political information processing. Political Psychology 38: 179–199.

[bibr30-09636625251325453] Kim S Capasso A Ali SH Headley T DiClemente RJ Tozan Y (2022) What predicts people’s belief in COVID-19 misinformation? A retrospective study using a nationwide online survey among adults residing in the United States. BMC Public Health 22(1): 1–12. 36401186 10.1186/s12889-022-14431-y PMC9673212

[bibr31-09636625251325453] King CM McCashin D (2022) Commenting and connecting: A thematic analysis of responses to YouTube vlogs about borderline personality disorder. Internet Interventions 28: 100540. 35493438 10.1016/j.invent.2022.100540 PMC9048063

[bibr32-09636625251325453] Koerber R Jennings MB Shaw L Cheesman M (2017) Representations of workers with hearing loss in Canadian newspapers: a thematic analysis. International Journal of Audiology 56(4): 260–266. 27967271 10.1080/14992027.2016.1265155

[bibr33-09636625251325453] Kozyreva A Lewandowsky S Hertwig R (2020) Citizens versus the internet: Confronting digital challenges with cognitive tools. Psychological Science in the Public Interest 21(3): 103–156. 33325331 10.1177/1529100620946707 PMC7745618

[bibr34-09636625251325453] Krange O Kaltenborn BP Hultman M (2021) “Don’t confuse me with facts”—how right wing populism affects trust in agencies advocating anthropogenic climate change as a reality. Humanities and Social Sciences Communications 8(1): 255.

[bibr35-09636625251325453] Lakomý M Hlavová R Machackova H (2019) Open science and the science-society relationship. Society 56: 246–255.

[bibr36-09636625251325453] Lee C (ed.) (1992) Proceedings: the First National People of Color Environmental Leadership Summit. New York: United Church of Christ Commission for Racial Justice.

[bibr37-09636625251325453] Lefere S Verghote K De Bruyne R Provoost V Satalkar PP (2023) ‘A radical operation’–a thematic analysis of newspaper framing of bariatric surgery in adolescents. BMC Public Health 23(1): 447. 36882787 10.1186/s12889-023-15366-8 PMC9993750

[bibr38-09636625251325453] Lewandowsky S Oberauer K (2016) Motivated rejection of science. Current Directions in Psychological Science 25(4): 217–222.

[bibr39-09636625251325453] Lewis D Keenan EL (1975) Adverbs of quantification. In: Keenan EL (ed.) Formal Semantics of Natural Language. Cambridge: Cambridge University Press, pp. 3–15.

[bibr40-09636625251325453] Lobato EJ Zimmerman C (2018) Examining how people reason about controversial scientific topics. Thinking & Reasoning 18: 1–25.

[bibr41-09636625251325453] Mellish TI Luzmore NJ Shahbaz AA (2020) Why were the UK and USA unprepared for the COVID-19 pandemic? The systemic weaknesses of neoliberalism: a comparison between the UK, USA, Germany, and South Korea. Journal of Global Faultlines 7(1): 9–45.

[bibr42-09636625251325453] Moorhead LL Fleerackers A Maggio LA (2022) “It’s my job”: A qualitative study of the mediatization of science within the scientist-journalist relationship. Journal of Science Communication 22: A05.

[bibr43-09636625251325453] Motta M Callaghan T (2020) The pervasiveness and policy consequences of medical folk wisdom in the US. Scientific Reports 10(1): 10722. 32612260 10.1038/s41598-020-67744-6 PMC7329847

[bibr44-09636625251325453] Motta M Callaghan T Sylvester S (2018) Knowing less but presuming more: Dunning-Kruger effects and the endorsement of anti-vaccine policy attitudes. Social Science & Medicine 211: 274–281. 29966822 10.1016/j.socscimed.2018.06.032

[bibr45-09636625251325453] Muringai V Fan X Goddard E (2020) Canadian consumer acceptance of gene-edited versus genetically modified potatoes: A choice experiment approach. Canadian Journal of Agricultural Economics/revue Canadienne D’agroeconomie 68(1): 47–63.

[bibr46-09636625251325453] Naeem M Ozuem W Howell K Ranfagni S (2023) A step-by-step process of thematic analysis to develop a conceptual model in qualitative research. International Journal of Qualitative Methods 22: 16094069231205789.

[bibr47-09636625251325453] Nisbet MC Scheufele DA (2009) What’s next for science communication? Promising directions and lingering distractions. American Journal of Botany 96(10): 1767–1778. 21622297 10.3732/ajb.0900041

[bibr48-09636625251325453] Nogara G Vishnuprasad PS Cardoso F Ayoub O Giordano S Luceri L (2022) The disinformation dozen: An exploratory analysis of covid-19 disinformation proliferation on twitter. In: Proceedings of the th14 ACM Web Science Conference, 2022, pp. 348–358.

[bibr49-09636625251325453] Nyhan B Reifler J (2019) The roles of information deficits and identity threat in the prevalence of misperceptions. Journal of Elections. Public Opinion and Parties 29(2): 222–244.

[bibr50-09636625251325453] O’Connell C McKinnon M LaBouff J (2020) One size does not fit all: gender implications for the design of outcomes, evaluation and assessment of science communication programs. JCOM 19(01): A06.

[bibr51-09636625251325453] Olson S (Ed) (2018) The science of science communication III: Inspiring novel collaborations and building capacity: Proceedings of a Colloquium. National Academies Press. 29901953

[bibr52-09636625251325453] Peters HP (2013) Gap between science and media revisited: Scientists as public communicators. Proceedings of the National Academy of Sciences 110(suppl. 3): 14102–14109. 10.1073/pnas.1212745110 PMC3752168 23940312

[bibr53-09636625251325453] Philipp-Muller A Lee SW Petty RE (2022) Why are people antiscience, and what can we do about it? Proceedings of the National Academy of Sciences 119(30): e2120755119. 10.1073/pnas.2120755119 PMC9335320 35858405

[bibr54-09636625251325453] Pjesivac I Hayslett MA Binford MT (2020) To eat or not to eat: Framing of GMOs in American media and its effects on attitudes and behaviors. Science Communication 42(6): 747–775.

[bibr55-09636625251325453] Rekker R (2021) The nature and origins of political polarization over science. Public Understanding of Science 30(4): 352–368. 33594929 10.1177/0963662521989193 PMC8114456

[bibr56-09636625251325453] Rutjens BT Heine SJ Sutton RM van Harreveld F (2018) Attitudes towards science. Advances in Experimental Social Psychology 57: 125–165.

[bibr57-09636625251325453] Said N Fischer H Anders G (2021) Contested science: Individuals with higher metacognitive insight into interpretation of evidence are less likely to polarize. Psychonomic Bulletin & Review: 1–13. 10.3758/s13423-021-01993-y PMC8555729 34716563

[bibr58-09636625251325453] Schlosberg D (2013) Theorising environmental justice: the expanding sphere of a discourse. Environmental Politics 22(1): 37–55.

[bibr59-09636625251325453] State of Science Index: Global Report (2023) Available at: https://www.3m.com/3M/en_US/3m-forward-us/about-the-survey/ (accessed 26 March 2023).

[bibr60-09636625251325453] Stocking SH Holstein LW (2009) Manufacturing doubt: Journalists’ roles and the construction of ignorance in a scientific controversy. Public Understanding of Science 18(1): 23–42. 19579533 10.1177/0963662507079373

[bibr61-09636625251325453] Stokel-Walker C (2023) Why is Twitter becoming X? New Scientist 259(3): 449.

[bibr62-09636625251325453] Sturgis P Allum N (2004) Science in society: re-evaluating the deficit model of public attitudes. Public Understanding of Science 13(1): 55–74.

[bibr63-09636625251325453] Sumner P Vivian-Griffiths S Boivin J Williams A Bott L Adams R Chambers CD (2016) Exaggerations and caveats in press releases and health-related science news. PLoS One 11(12): 0168217. 10.1371/journal.pone.0168217 PMC5158314 27978540

[bibr64-09636625251325453] Terry G Hayfield N Clarke V Braun V (2017) Thematic analysis. SAGE Handbook of Qualitative Research in Psychology 2(17-37): 25.

[bibr65-09636625251325453] Van Scoy LJ Snyder B Miller EL Toyobo O Grewal A Ha G , et al (2022) ‘Us-versus-them’: Othering in COVID-19 public health behavior compliance. PLoS ONE 17(1): e0261726. 10.1371/journal.pone.0261726 PMC8786185 35073346

[bibr66-09636625251325453] Ward JK (2019) Boundary-making in the media coverage of the 2009 pandemic flu vaccine’s safety in France. Journalists and Science 10: e0219.

[bibr67-09636625251325453] Weisberg DS Landrum AR Hamilton J Weisberg M (2021) Knowledge about the nature of science increases public acceptance of science regardless of identity factors. Public Understanding of Science 30(2): 120–138. 33336623 10.1177/0963662520977700

[bibr68-09636625251325453] WHO (2023) Statement on the Fifteenth Meeting of the IHR Emergency Committee on the COVID-19 Pandemic, 5 May. Available at: https://www.who.int/news/item/05-05-2023-statement-on-the-fifteenth-meeting-of-the-international-health-regulations-(2005)-emergency-committee-regarding-the-coronavirus-disease-(covid-19)-pandemic

[bibr69-09636625251325453] Wojcik S Hughes A (2019) Sizing up Twitter users. PEW Research Center 24: 1–23.

[bibr70-09636625251325453] Wright S (2018) The impact of “super-participants” on everyday political talk. Journal of Language and Politics 17(2): 155–172.

[bibr71-09636625251325453] Zhou Y Shen L (2022) Confirmation bias and the persistence of misinformation on climate change. Communication Research 49(4): 500–523.

