# Supplementary material for: Contested science communication: Representations of scientists and their science in newspaper articles and the associated comment sections
Source: Public Underst Sci. 2025 Mar 17;34(6):810–28. doi: 10.1177/09636625251325453 (PMC12274558; doi:10.1177/09636625251325453)
Supplement: sj-docx-1-pus-10.1177_09636625251325453 – Supplemental material for Contested science communication: Representations of scientists and their science in newspaper articles and the associated comment sections [file sj-docx-1-pus-10.1177_09636625251325453.docx]

**Supplemental Material**

The following provides information about the authors and supplemental material of 1) more (anonymized) examples from the text corpus with comments and codings, 2) a list of the codes, and 3) the data distribution showing the articles sorted into four categories: News, News (Opinion piece), Editorial, and Column. Additionally, this list shows the type of author (e.g., journalist, editor, or columnist).

**Authors**

1. Katrine K. Donois

<https://www.aru.ac.uk/people/katrine-kergrohen-donois>

Katrine is a PhD researcher in the Faculty of Science and Engineering at Anglia Ruskin University. Katrine's research concerns the multiple ways in which science is communicated or understood and examines the processes that impact effective communication.

1. Dr. Lewis Goodings

<https://www.aru.ac.uk/people/lewis-goodings>

Lewis is a qualitative social psychologist who is interested in the way that self and identity are mediated via social media technologies. He also considers the use of these technologies in relation to mental distress and the possible advantages or disadvantages therein.

1. Professor Mick Finlay

<https://www.aru.ac.uk/people/mick-finlay>

Mick carries out research into political communication, group conflict, and intellectual disabilities and teaches Social Psychology and Qualitative Research Methods. He is a Fellow of the Higher Education Academy and leader of the Empowerment and Social Justice Research Group at ARU.

1. Dr. Nicola Gibson

<https://www.aru.ac.uk/people/nicola-gibson>

Nic is interested in Applied Psychology, particularly well-being, resilience, and pro-sociality as personal strengths that influence positive outcomes. Research areas include play and playfulness in adults, human-animal companionship, gaming, and individual differences.

**Supplemental material: More (anonymized) examples from the text corpus with comments and codings**

(Important parts of the quotes and comments were highlighted. Thoughts were noted, and codes were added (they can be found beneath each quote/comment). Please note that the comments are presented in their original form, which includes spelling errors.)

**Article, Climate Change:**

“For decades, public health, weather and climate experts have grappled with how to raise public awareness of heat waves and the understanding of their threat.”

Thoughts: Experts have grappled for decades with how to relay the threat of heat waves. Can’t figure out how?

Code(s): Urgent attention, Threats from Nature (good/bad Nature)

**Comment, Climate Change:**

“[...] This forum is over run with quacks and cranks with virtually zero knowledge of the relevant subjects but who feel their ignorance is proof they can attack the experts with obnoxious certainty.”

Thoughts: Angry comment (fed up?). Dunning-Kruger effect?

Code(s): Normal vs. Crazy, Ignorance

**Comment with reply, Climate Change:**

**Comment**:

Yes another apocalyptic piece accompanied with a dramatic picture of a CA wildfire. Climate change and the magical powers of CO2 really are a religion for some. The earth's climate has always varied and it occurs naturally. It warms up and it cools down. Man's impact on the climate is negligible. There is nothing extraordinary about the weather, it is cyclic. Good grief, they (the climatologists) can't predict the weather for a holiday weekend never mind next decade.

Thoughts: Media hype. A religion to some. Downplays impact. “Climate change” versus “weather.”

Code(s): Blame (Media). Scientists don’t know.

**Reply:**:

*Climate change and the magical powers of CO2 really are a religion for some.*

You mean the deniers who think they know better than climate scientists.

*Good grief, they (the climatologists) can't predict the weather for a holiday weekend never mind next decade.*

But you think that you with no expertise can say that putting many gigatons of a greenhouse gas into the atmosphere every year will have a negligable effect.

Thoughts: Knowing better than experts (arrogance?). Sarcastic reply.

Code(s): Blame (ignorance)

**Article, Vaccines:**

“Now, health experts have warned that even if a coronavirus vaccine is approved, refusals could open the way to a resurgence of the virus while threatening efforts to keep other preventable diseases in check.”

Thoughts: The situation is described as worrisome.

Code(s): Scientists: Worried. Prevention.

**Comment, Vaccines:**

“Do you think maybe it's a trust issue? Certain groups have sown so much distrust of governments, politicians, and medicine, that there seems to be too many choices for people to make. If you don't trust those that are telling you the truth, you have to make up your own mind. There is just so much social media and conspiracy theorists, that I can get a different answer to the same question ten times a day.”

Thoughts: Different format, begins with a question. Could be a rhetorical question? Appears to try to engage in debate. Highlights trusts, too many choices, social media + conspiracy theorists.

Code(s): Trust. Internet.

**Comment, Vaccines:**

“I do not believe that the industry is honest with us. I do not believe that we are told of the possible side effects. I believe that science and commercial interest are in conflict and that this dangerous situation has now been propagated well beyond the field of medicine into food production. I no longer believe scientists are honest.

Thoughts: Working for industry, selling products - impact on trust in scientists

Code(s): Blame: Industry. Trust. Blame: Money. Unintended consequences/dangerous science

**Article, GMOs:**

“Nonetheless, gene modification scientists are focusing increasingly on building health benefits into widely used foods. In addition to pink pineapples containing the tomato-based antioxidant lycopene, tomatoes are being engineered to contain the antioxidant-rich purple pigment from blueberries.

And people in developing countries faced with famine and malnutrition are likely to benefit from attempts to improve the protein content of food crops, as well as the amount of vitamins and minerals they provide.”

Thoughts: GMOs are described as something that can be used in relation to health benefits + aid developing countries faced with famine and malnutrition.

Code(s): The good guys (saving, fixing, solving, helping)

**Comment, GMOs:**

“We have gotten along without golden rice for some 66 million years. And it is more wicked for scientists to continue to raise the carrying capacity of the earth than to simply allow our population levels to adjust to a sustainable carrying capacity.”

Thoughts: We don’t need this. Helping/saving people is a problem (sustainable carrying capacity).

Code(s): Why?Bad guys/mad scientist

**Comment, GMOs:**

“More anti-science stupidity from the uneducated masses who think they are smarter than scientists. Exactly the same phenomenon as climate denial (although the latter disease is more prevalent on the right, the former perhaps more prevalent on the left).

This is not merely academic: both climate denial and GMO phobia cost lives. Sad.

Thoughts: Hints at politics. Equals Cli. Cha. denial and anti-GMOs to disease. GMO phobia costs lives.

Code(s): Blame: Ignorance. Blame: Politicians/Politics. Anti-science ruins lives/world

**Supplemental material: List of codes**

| **Themes** | **Codes**  **(Newspaper articles)** | **Codes**  **(Reader Comments)** |
| --- | --- | --- |
| **Theme 1: Representations of the scientist behind the science: Establishing trustworthiness and credibility through references to quality.** | Quality of science  Expert knowledge - Expertise  Consensus  Mainstream/real science  Trustworthiness  Credibility  Truth | Quality of science  Real science  Junk science  Experts  Evidence  Consensus  Credibility  Trust/Trustworthiness |
| **Theme 2: Heroes or villains: Conflicting representations of scientists and their motives.** | The good guys (saving, fixing, solving, helping)  Prevention  Advocacy  Saving  Danger (not listening to scientists)  Scientists: Worried  Scientists: Angry  Scientists: Frustrated  Ethics/Morality | The good guys (saving, fixing, solving, helping)  Anti-science ruins lives/world  Bad guys/mad scientist  Arrogance  Hubris  Playing God  Unintended consequences/dangerous science  Ethics & Morality |
| **Theme 3: The unnatural nature of science: The positioning of scientists in relation to Nature and folk wisdom.** | Urgent attention  Threats from Nature (good/bad Nature)  Safety of science  Helping (Mother) Nature  Risks | Urgency  Threat to Nature: Threatened by science  Fear of science  Safety  Risk  Why?  Good Nature  Mother Nature  Folk wisdom |
| **Theme 4: Reasons for rejecting expertise and science: Accountability, blame, and lack of debates.** | Science communication  Listen to experts  Science not debatable  Normal vs. Crazy  Respect  Support  Science as a job    Blame:  *Activist groups*  *Misinformation*  *Ignorance*  *Opposition*  *Politicians/Politics*  *Inaction*  *Contrarians/skeptics/deniers*  *Internet*  Trust | Being wrong (accountability)  Right to ask questions  Right to know(ledge)  Right to choose  Science communication  Call for debate  (Dis)Respect  Normal vs. Crazy  Blame:  *Ignorance*  *Inaction*  *Science as a job*  *Industry*  *Money*  *Power*  *Media*  *Politicians/Politics*  *Misinformation*  *Internet*  Trust  Scientists don’t know |

**Supplemental material:**

**Data distribution**

Below is a list showing the articles sorted into four categories: 1) News, 2) News (Opinion piece), 3) Editorial, and 4) Column. Additionally, the list shows the type of author (e.g., journalist, editor, or columnist).

The distribution is as follows:

84 articles in total.

1) News: 55 articles

2) News (Opinion piece): 10 articles

3) Editorial: 5 articles

4) Column: 14 articles

**GMOs**

**The Guardian**

| Article 1 News | Journalist |
| --- | --- |
| Article 2 News | Journalist |
| Article 3 News (Opinion piece) | Contributing Opinion Writer |
| Article 4 News | Journalist (Science Editor) |
| Article 5 Editorial | Observer Editorial |
| Article 6 News | Journalist |
| Article 7 News | 2 x Journalists |

**The Times**

| Article 1 Column | Columnist |
| --- | --- |
| Article 2 News | Journalist |
| Article 3 Column | Columnist |
| Article 4 News | Journalist |
| Article 5 News | Journalist |
| Article 6 News | Commentator |
| Article 7 News (Opinion piece) | Contributing Opinion Writer |

**The Washington Post**

| Article 1 News | Journalist (Reporter) |
| --- | --- |
| Article 2 News (Opinion piece) | Contributing Opinion Writer |
| Article 3 News (Opinion piece) | Contributing Opinion Writer |
| Article 4 News (Opinion piece) | Contributing Writer (Consumer Reports) |
| Article 5 News (Opinion piece) | Contributing Opinion Columnist |
| Article 6 News | Journalist |
| Article 7 Column | Columnist |

**The New York Times**

| Article 1 News | Journalist |
| --- | --- |
| Article 2 Column | Columnist |
| Article 3 News | Contributing Writer |
| Article 4 News | Journalist (reporter) |
| Article 5 News | Contributing Writer |
| Article 6 News | Journalist |
| Article 7 News | Journalist |

**Climate Change**

**The Guardian**

| Article 1 News | Contributing Writer |
| --- | --- |
| Article 2 News | Contributing Writer |
| Article 3 News | Journalist (editor) |
| Article 4 News (Opinion piece) | Contributing Opinion Writer |
| Article 5 News (Opinion piece) | Contributing Opinion Writer |
| Article 6 News | Contributing Writer |
| Article 7 News | Journalist (editor) |

**The Times**

| Article 1 Column | Columnist (journalist) |
| --- | --- |
| Article 2 News | Journalist |
| Article 3 News | Journalist (editor) |
| Article 4 News | Journalist (editor) |
| Article 5 News | Journalist (US West Coast bureau chief) |
| Article 6 News | Journalist |
| Article 7 News | 2 x Journalists |

**The Washington Post**

| Article 1 News | Journalist |
| --- | --- |
| Article 2 News | Reporter |
| Article 3 News | 2 x Reporters |
| Article 4 News | 1x Reporter, 1x Editor |
| Article 5 News | Reporter |
| Article 6 News | Reporter |
| Article 7 Editorial | Editor |

**The New York Times**

| Article 1 News | Reporter |
| --- | --- |
| Article 2 News | Journalist |
| Article 3 News | 2 x Reporters |
| Article 4 News | 1x Reporter 1x Correspondent |
| Article 5 News | 2x Reporters 1x Correspondent |
| Article 6 News | Journalist |
| Article 7 News | Science Writer |

**Vaccines**

**The Guardian**

| Article 1 News (Opinion piece) | Contributing Opinion Writer |
| --- | --- |
| Article 2 News | Contributing Writer |
| Article 3 Column | Columnist |
| Article 4 Column | Columnist |
| Article 5 Column | Columnist |
| Article 6 News (Opinion piece) | Contributing Opinion Writer |
| Article 7 Column | Columnist |

**The Times**

| Article 1 News | 1x Journalist 1x Editor |
| --- | --- |
| Article 2 News | Contributing Writer |
| Article 3 News | Correspondent |
| Article 4 News | Journalist |
| Article 5 News | Correspondent (journalist) |
| Article 6 News | Journalist |
| Article 7 Column | Columnist |

**The Washington Post**

| Article 1 News | Reporter |
| --- | --- |
| Article 2 News | 1x Reporter 1xJournalist |
| Article 3 Editorial | Editor |
| Article 4 News | Reporter |
| Article 5 News | Contributing Writer |
| Article 6 News | Contributing Writer |
| Article 7 Column | Columnist |

**The New York Times**

| Article 1 News | Reporter |
| --- | --- |
| Article 2 Column | Opinion Columnist |
| Article 3 Editorial | Editorial Board (opinion journalists) |
| Article 4 Column | Columnist |
| Article 5 Column | Columnist |
| Article 6 News | Audience writer for the New York Times |
| Article 7 Editorial | Editor |
